# Some Perils of Child-Bearing and Their Prevention

**Published:** 1898-05

**Authors:** Arthur E. Spohn

**Affiliations:** Corpus Christi, Texas


### TEXAS MEDICAL JOURNAL.

ESTABLISHED JULY, 1885.

PUBLISHED MONTHLY.—SUBSCRIPTION $2.00 A YEAR.

VOL. XIII. AUSTIN, MAY, 1898. No. 11.

### Original Contributions.

For the Texas Medical Journal.

### Some Perils of Child-Bearing and Their Prevention.

BY ARTHUR E. SPOHN, M. D., CORPUS CHRISTI, TEXAS.

[Read before the Central Texas Medical Association, January, 1897.]

When I accepted the invitation to write a paper for this Association on the prevention of diseases produced by child-bearing, I did not realize the magnitude of the subject, and the responsibility connected with it. It is impossible, in a short paper, to enter into detail, and the best I can do will be to erect, as it were, here and there, a landmark in places of extreme danger, and fix in our minds conditions demanding prompt decision and action, for the life of a fellow-being is at stake and often hangs upon a very short space of time. This paper will include a much greater scope than I at first anticipated—for diseases of child-bearing women are so intimately connected with and follow certain pathological conditions associated with the diseases of early conception, that a good knowledge of them all is quite important; and proper means adopted for their relief often prevent disastrous results during the puerperal state, for a diseased organ always performs its functions in an abnormal manner.

I have drawn almost entirely from my own personal experience, and where I fail to properly elucidate this great subject, I trust any deficiencies will be replaced by the discussion—for it is only by discussion that we can arrive at truths and establish fixed rules for our guidance.

Following the sin of Adam and Eve in the Garden of Eden, came the edict pronounced against woman by her Creator, "I will greatly multiply thy sorrow and thy conception; in sorrow thou shalt bring forth children." In course of time, because of the sin of the world, it was destroyed; but Noah found grace in the eyes of the Lord, and with his family, was saved and commanded to "increase, multiply and replenish the earth." And we would naturally infer that the burden of sorrow had been at the same time removed from woman, and look upon conception and parturition as a physiological process, the accompanying suffering not confined to mankind, but the whole animal kingdom. Were the sorrows and pain of child-bearing a punishment bestowed upon woman by her Creator, and could we believe in the divinity of such suffering we might naturally hesitate; no doubt for this reason, many have hesitated in attempting to ameliorate her condition. From generation to generation she has borne her suffering, in many instances superstitiously believing it a recompense for the sin of her mother Eve, with fortitude, down through the ages, until we find her at the close of the nineteenth century still willing and ready to descend to the very portals of death in order to give life. So closely is the desire for offspring intermingled with her very existence, that we find when Rachel discovered she was barren she besought her God, and "God hearkened to her and opened her womb and she conceived;" and in her great joy said, "God hath taken away my reproach." Little did Rachel suppose her life was to be given for her son Benjamin; and her case is the first reported of death from child-birth, and the first instance of the employment of a midwife—and justification of the operation of dilatation for sterility. In order to appreciate this subject it is necessary to study carefully the anatomy and physiology of the female organs of generation; and the effect and changes produced by inflammatory conditions of whatever cause, when the pathology and symptoms will be easily understood. Given a healthy woman, surrounded by good hygienic conditions, child-bearing should be a normal physiological process, never terminating fatally. Such might have been the case in the earlier centuries, but with existing civilization we may almost state, as a rule, few women are physically sound—distortions from tight lacing, misplaced organs, and changes in the organs themselves, chronic inflammations, specific or otherwise, indiscretions during menstruation, with numerous other subjective causes entirely out of the province of the physician to control or remedy. But there are causes which may be classed preventive, and these I will specially refer to in this paper; also refer to some unavoidable causes, such as perverted conditions, aborted processes, etc. The former including septic infection and mechanical injuries; and the accidents during the early months of pregnancy—in their relation to and effect upon the child-bearing woman, will be considered—not taking into consideration unavoidable causes, such as chronic kidney, tuberculous, cancerous, or other incurable disease. I do not think I can do better to illustrate this subject than by following a case from conception to parturition. The husband and wife are healthy—she misses a monthly period, and the first obstacle she encounters will be nausea, which may be ameliorated by the ordinary means in general use, and an assurance that it will cease in a certain time. Sometimes elevating the uterus with a soft pessary will relieve pressure; but it must be remembered that there are cases when all means will fail, and we must not delay too long in emptying the uterus of its contents—I have been forced to this procedure in several instances—and once where the patient had a large quantity of albumin in the scanty urine, and she suddenly developed the most intense convulsions. This will show the necessity of examining the urine from time to time, even in the early months of pregnancy. I would not recommend emptying the uterus until all recognized remedies had failed, yet it may be better than defer until the case becomes desperate, for we have the chances of abortion, etc. Some advise the necessity of emptying the uterus should be suggested, when there is the presence of coffee grounds material in the vomit—sub-sternal pain and distress with marked anæmia and emaciation. The most persistent vomiting and distress I ever witnessed occurred a month ago in a young lady, primipera, at the eighth month, and after other physicians, as well as myself, had tried every means she miscarried, a five or six months fœtus, after great exhaustion and weeks of suffering—here certainly interference would have saved much trouble.

Pain and hemorrhages in the early months must not be treated lightly, and demand prompt attention. Some women menstruate, or rather have periodical discharges of blood during the first few months, even during the whole time; these must not be confounded with hemorrhages, generally irregular and unexpected, and for this reason it is important that discharges of any kind during pregnancy demand consideration, for they are generally the result of aborted or perverted conditions which may prove most disastrous. There may be slight discharges of blood due to local affections of the cervix, such as polypoid growth, laceration with erosion and cancer, which are easily detected, but the condition most alarming is pain with hemorrhage. After trying all usual remedies, if the os becomes dilated with protruding membranes, we must aid in the expulsion of uterine contents, for in such cases abortion is unavoidable. The case may be one of missed abortion, the ovum is dead and retained; here we have danger from septic infection or the ovum may continue as a mole—or the abortion may be incomplete, portions remaining become offensive. In all such cases, or when in doubt, a thorough examination must be made, dilating if necessary, using finger and curette to remove contents, under strictly antiseptic precautions. I once found the placenta in a case of incomplete abortion compressed and flattened like a button, held in the fundus of the uterus; it was easily detected by the finger, curetting would certainly have failed to remove it. In molar pregnancy the ovum is apoplectic or fleshy—compressed, and the uterus smaller than it should be at the corresponding period of gestation. The trouble is caused by the existence of a foreign body, with pain and hemorrhage. In hydatidiform degeneration the uterus will be larger than the corresponding period, with pain, hemorrhage and discharge of vesicles. In all such cases ergot may be tried first; if that fails, rapid dilation and removal of contents will be necessary. In decidual endomentritis, we have pregnancy occurring during subinvolution, or chronic endometritis, the uterus becomes large with occasional gushes of serum which may be colored with blood. This condition may occur in cases of syphilis, and under the use of potassium iodide may go to full term, however all such cases generally abort. I remember one case where the lady lost the child at seventh month under such circumstances four times; another who carried a dead fœtus until she accidentally detected a bone in her vagina. In all such cases the greatest antiseptic precautions must be used, and in curetting a dull instrument is preferable. If sharp, the blade must be directed backwards, for the uterine walls are quite soft and easily injured. I am much in favor of uterine irrigations of zinc sulphate grs. ½ to the ounce, following antiseptic washes, corrosive sublimate, per oxide of hydrogen, formol, etc., with an occasional application of glycerite of lead; our object being to restore the uterus to as normal a condition as possible. There is liable to be adherent placenta or portions of it—with danger of post partum hemorrhage.

The most important of all early hemorrhages will be found in cases of extrauterine pregnancy—rare, yet if mistaken generally terminating fatally. The woman may miss one or two periods, or has an irregularity in her menstruation, suddenly she is seized with intense pain in either ovarian region, or behind the uterus, accompanied by hemorrhage and an unusual discharge of debris, at times deciduous membrane. If rupture has taken place, you will, as a rule, have the symptoms of hemorrhage, cold perspiration, great exhaustion, dimness of vision, a rapid pulse, anxious expression, a general state of collapse. She will have difficulty in extending the limb of the affected side, with a sense of tension and fullness, a desire to evacuate bladder and bowels. An examination reveals a soft uterus, with a mass on either side or behind. There is but one remedy, she is bleeding to death, and the artery supplying the blood must be tied.

There is no case more tests the skill,
The steady nerve, the power of will,
Than where, from causes now unknown,
A living ovum has been thrown
Into the tube, is fixed and grows,
And by increasing slow, so slow,
That tube no longer can contain
Its living contents, bursts, in twain is rent,
The life blood flows from unclosed vent,
The mother sinks, her eyes grow dim,
All hope seems lost. Hold! See, comes in,
A noble mind, the bravest heart,
A steady hand well trained in art;
He ope's the wall, a string is laid
Around the tube, her life is saved.
There is no time so sure we stand,
Holding a fellow life in hand,
Decision, acting well our part,
Steady of nerve and brave of heart,
Doing no more than should be done
To our own selves were we the one.

If the rupture is in the broad ligament, the tumor small, no urgent symptoms, we may wait; but rupture in the ligament is rare, and when it does take place, secondary rupture into the peritoneal cavity soon follows, with severe hemorrhage, so that as soon as these cases are detected, the better plan is to open the abdomen at once, ligate, and remove the tube and sac. I

will not consider vaginal operations for this condition. In many cases where attempted it was found necessary to open the abdomen above also, to ligate and control the hemorrhage. Placenta prævia is another cause of hemorrhage, generally commencing between the fourth and sixth months. The flow will be irregular, sometimes but little, then coming in gushes. Later on it may be so severe as to require interference, with threatened collapse. Dilatation is best made with the finger—first one and then two, etc., under chloroform, and the child delivered—of course circumstances with determine the necessity for active interference.

From the sixth month to the termination and even a few days after parturition the patient may have puerperal eclampsia, and, as I stated before, an occasional examination of the urine should be made, more especially if there is any swelling of the limbs, eyelids, headache, etc.; whatever may be the cause of puerperal eclampsia, it is always accompanied with albumin in the urine, and most severe when the urine is scanty and albumin excessive. Upon the appearance of symptoms, the bowels should be regulated with salines, a careful diet ordered, principally milk, broth, etc., and the functions of the skin stimulated, warm baths, or hot air baths are beneficial, and sometimes allowing the patient to assume positions so as to avoid pressure on the kidneys—lying on the side has been recommended. The urine may contain granular and hyaline casts. Should urgent symptoms appear, small doses of pilocarpin may be given, or veratrum viride, with digitalis. If convulsions supervene, venesection will be in order; in a strong patient twenty or thirty ounces of blood may be drawn. Chloral is sometimes useful. Pilocarpin should be injected hypodermically, $\frac{1}{10}$ to $\frac{1}{6}$ of a grain every two hours—omit if symptoms of œdema of lungs appear—with veratrum viride tr., three to five minims by hypodermic injection every hour, until three or four doses have been given. I have on several occasions controlled the convulsions by hypodermic injections of pilocarpin, grs. $\frac{1}{6}$; digitalin $\frac{1}{100}$ gr., morphia and atropia, $\frac{1}{4}$ and $\frac{1}{150}$ gr. I believe morphine is best, the object being to quiet, lessen blood pressure, stimulate the action of the skin, and increase the action of the kidneys, at the same time the cervix should be dilated and labor induced. I have found nothing better than the constant pressure of one finger, then two, etc. After labor, great care must be taken to prevent sepsis. Parts are cleaned, vagina irrigated, also uterus, with 1 to 3000 corrosive sublimate, or boric acid solution, or peroxide of hydrogen—in fact, all may be used if so desired; lately I prefer weak solutions of formol.

In septic infection as a cause of disease in child-bearing women, we have an important factor, which in part is under control. In all our examinations and operations, surroundings should be *clean*, not in the general meaning of the word, but surgically clean; room, bedding, etc., a nurse who is not menstruating at the time, with her person and clothing clean. The patient is prepared as carefully as if an abdominal section were to be made, and if a case of labor, the bowels should be regulated, and an injection given at the commencement, to remove anything remaining in the rectum, and care taken that the bladder is empty. She may have a vaginal douche, five per cent. boracic acid solution, or formol solution, in sterilized water, first carefully cleaning the external genitalia, irrigating the vulva, then vagina, If not carefully and properly done it is much better not to give it. If necessary to use a catheter, the instrument must be boiled, the vulva separated and irrigated, and the instrument inserted by direct inspection. The method of doing the modest act under cover, gathering the germs in the introitus, forcing them against the meatus with the end of the finger then pushing them into the bladder, is abominable. The physician must also be specially prepared, clean clothing, carefully cleaning his hands, using soap and nail brush freely. Dr. Joseph Price recommends mustard for the hands, well rubbed in while in the process of cleaning—he thinks it renders the skin softer and makes the sense of touch more delicate, while the oil of mustard is an excellent antiseptic. Thus carefully prepared, we are ready to make an examination if necessary, and should never lose sight of the fact that we are dealing with one willing to lay down her life for another, who should receive every consideration.

The first examination will determine the condition of the pelvic cavity, presentation, etc., and if the labor is normal, no further examination is necessary; for "meddlesome midwifery is bad." Those who are constantly making examinations, and as it were, trying to help their patients, generally succeed in helping them—out of the world. A plain statement that everything is going on well, and unnecessary attention bad, will generally give satisfaction. Everything tending to make her comfortable should be tried, sheets carefully adjusted, soiled clothes removed, and a thousand little attentions I need not mention. A couple of sheets thrown over the limbs so as to expose the parts without her knowledge, will enable the nurse to watch the progress from time to time; and when the vulva is advancing, the physician may assume such position as will enable him to render assistance when necessary. The membranes, as a rule, should not be ruptured—and if this rule is followed, the soft parts will suffer much less from pressure, we will have fewer injuries and lacerations to repair, and lessen the tendency to many conditions affecting the future health and comfort of the mother. Chloroform may be given, but I think women do much better without any anesthetic--still am in the habit of giving it if the suffering is great and they so desire. After the child is born, the physician's hand may rest gently over the abdomen, covering the uterus, which will enable him to determine its condition. Gentle intermittent pressure will stimulate the uterus to contract in a few minutes, when the placenta may be removed. The parts are now carefully cleaned, the vagina irrigated with five per cent Boric Acid sol., or Formol, and the parts carefully examined for lacerations. Do not put confidence in what writers term "physiological lesions," because they absorb septic materials, and the dense lymphatic connection will soon give you trouble you did not anticipate. If they are found, a roll of cotton is passed above into the vagina, the part irrigated and every lesion appropriately closed. I cannot enter into detail, but merely state what has seemed important in my own experience.

Now it really seems out of place to place so much importance in cleanliness—just as if all physicians were not careful and clean. Are they? Do they all consider what a vast absorbing surface is exposed, continuous with the peritoneal cavity? Would they all be willing to let the rays of a search light fall on their methods of obstetrical practice? I think not. I have seen the most eminent members of the profession drive up to a house, tie their horse, walk in and make a careful vaginal examination without washing their hands; and when they do so, if they do not drive a nail in that woman's coffin, it is no fault of theirs. Now septic infection in a healthy woman is caused by dirty surroundings, dirty person, dirty, ignorant, careless, slovenly doctors; and careless, dirty, ignorant nurses. What is the remedy? do you know? I think I do—simply doing right, no more. But there is another kind of septic infection, which, up to the present time, we have not been able to prevent. A pure, innocent young girl, the picture of health and beauty, "places her young and loving heart into the hands of him whom she loves;" everything seems bright and happy for her. In course of time she becomes pregnant, is eventually confined. In a few days you notice the child's eyelids swollen, glued together, with yellow pus emerging from the fissure. The mother has a pain in the uterine region, high fever, etc. You have been extremely careful in her case, and cannot account for such a furious septic infection. She dies; the child becomes blind, the nurse loses one or both eyes from the same cause (this occurred in my practice); you ask the husband if he ever had gonorrhœa; his answer is: "Yes, several years ago, but I was cured." That husband loved his wife, you cannot tell him that he unconsciously murdered her;—and so the innocent destroyer moves on, and you forget the circumstance until a case of sickness occurs in that family, and another physician is employed, and you are probably blamed for the previous misfortune. So much for your pains. Or the woman miscarries, has salpingitis, becomes an invalid. Abscesses form in tube or ovary, involving adjacent organs—after years of suffering, the gynecologist reaps a large fee and records one more successful section 5208, and a motherless woman spends the remainder of her days in sorrow. How can we prevent this? I don't know. We spend thousands of dollars annually in the prevention of contageous diseases; we quarantine strictly against yellow fever, small-pox, etc., and I am safe in stating that where one dies of either of those diseases, one hundred women die from gonorrhœal infection; another hundred made miserable for life, and numberless children blind. I often wonder if mothers, or women generally, know anything of all this—if they did, I believe such a crusade would take place as to shake the very foundations of society. When we examine these facts seriously, a problem is presented of such magnitude as to stifle all our efforts at sanitation; and with advancing civilization it increases, almost nil in the rural districts, but in railroad and steamboat towns, the number of cases of salpingitis and pustubes is alarming, not mentioning the subacute cases infecting parturient women. Dr. Lawson Tait recognized this fact years ago, stating: "We see more of the worst results of extension of the process in women, who have recently been confined. * * * ". In speaking of syphilis and gonorrhœa, he states: "Where syphilis kills its tens, gonorrhœa kills its thousands, and it would take the sufferings of a hundred cases of syphilis to make up for the long, weary years of agony of one case of gonorrhœal pyosalpinx;" and he has not been able to suggest a remedy. Can the Central Texas Medical Association suggest one? It appears to me that some legislation should be enacted to cover these conditions and give protection to innocent, confiding women. That no license to marry should be issued unless good evidence was produced that the party was in proper sanitary condition. In these cases of suspected gonorrhœal infection, great care must be taken to prevent septic trouble during child-birth. Copious injections of one to two or three thousand corrosive sublimate, or solution of formol may be used; the child's eyes carefully cleaned, and every precaution taken against infection of the large absorbing surface, necessarily exposed.

I do not claim that all cases of pustubes, etc., are caused by gonorrhoeal infection. We have a very potent factor in the uterine teaser, office gynecologists of the larger cities--five to twenty-five dollars for consultation. Embryonic professors who have become specialists six months after graduation, who probably never opened a boil or lanced a baby's tooth. Women flock to such men by hundreds, are probed, dilated, punctured, cauterized, etc., etc. The little uterus is an excellent incubator, you can't get a better one—by carelessness, dirty instruments, etc., by prolonged irritation subacute inflammation supervenes —extends to the tubes--I need not record the history following. After great and prolonged suffering, the operating gynecologist gets in his work, probably another operator—records section No. 3210. Or we are in the rural districts and the popular M. D. goes from house to house with his little black satchel; he walks in, greases his spec., washes his hands in a dirty basin, wipes on the most convenient towel, makes an examination, does the probing, dilating boro-glyceride act; charges five dollars for special attendance; woman passes from one doctor to another, suffers years, is a chronic invalid, ends on the operating table; successful section, 2500. Is all this true or not? I have no objection to specialists, on the contrary I consider them as occupying the most difficult position imaginable. There is no condition of mental strain and anxiety equal to that of the abdominal surgeon. Here a mistaken diognosis means something; here careless work means loss of life; here an opinion is verified by actual demonstration. What a careful preparation is necesssary—what prolonged years of study—what extensive and differential diognosis. These combined with honesty and conscientiousness, place a physician in a position specially adapted to claim consideration in any special branch of the profession he may select,—"Heights by great men gained and kept were not attained by sudden flight;" and thus fully prepared, when an unfortunate woman presents herself for examination and treatment, such methods are adopted as to give her the advantage of skilled attendance, be it lacerations, displacements, changes in the organs—from sub-involution, etc., giving due consideration to each and every condition, combined with mental and bodily rest; careful nursing, thus restoring the important organs of generation to a normal condition. In this way alone can the diseases peculiar to child-bearing women be prevented. After the birth of the child there are conditions requiring attention—one of the most important, post partum hemorrhage, generally caused by uterine inertia—or placental adhesion—retention of blood from stoppage of the cervix by clots. The uterus is felt very much enlarged as it were distended, with symptoms of loss of blood, which may be quite alarming. The best treatment is to excite uterine contractions, remove anything from the uterine cavity, keeping one hand over the uterus, making gentle pressure frictions, etc.,—adherent placenta must be removed, the hand is the best instrunent; then irrigate the cavity, give ergot, etc. Many drugs are recommended, some carry a sponge into the cavity, saturated with warm turpentine or turpentine and water. I have never tried this, in fact I have never seen a severe case of post partum hemorrhage. If the hemorrhage continues after the uterus has contracted, we must examine for lacerations and ligate the bleeding vessels if necessary. Much care must be used in adjusting bandages after childbirth. Some apply none. I am opposed to extremes in anything; a gently pressing bandage is grateful to the patient, and the one made of flannel torn on either side into strips three inches wide is preferable, adjusted like the many tail bandage. The upper and lower part of bandage may be applied firmly, but very little or no pressure over the uterus, avoiding forcing it out of position. This is quite important. The vulva should be covered with a pad of gauze or aseptic cotton, changed when soiled, and every precaution used as to cleanliness, and when diseased conditions are properly remedied, and care and attention given to the parturient woman, little is to be feared. The Preston Retreat at Philadelphia, during the administration of Dr. Joseph Price, out of 1400 cases of confinement did not have a single death. I asked Dr. Price the cause of such success, and his answer was—cleanliness. I might continue this paper indifinitely, but will close with a conversation I heard while in Philadelphia a few months ago: A medical friend of mine was going to the city, his little six-year-old daughter said: "Papa, take me to the city with you." "I can't my child, I have several operations." "Oh, you always have operations." "Do you know what an operation means?" "Yes, papa, fixing people's insides right."

